# Origin of Broad Emission Induced by Rigid Aromatic Ditopic Cations in Low-Dimensional Metal Halide Perovskites

**DOI:** 10.1021/acs.jpclett.3c01872

**Published:** 2023-08-28

**Authors:** Marta Morana, Waldemar Kaiser, Rossella Chiara, Benedetta Albini, Daniele Meggiolaro, Edoardo Mosconi, Pietro Galinetto, Filippo De Angelis, Lorenzo Malavasi

**Affiliations:** † Department of Chemistry and INSTM, University of Pavia, Via Taramelli 16, Pavia 27100, Italy; ‡ Computational Laboratory for Hybrid/Organic Photovoltaics (CLHYO), Istituto CNR di Scienze e Tecnologie Chimiche “Giulio Natta” (CNR-SCITEC), Perugia 06123, Italy; § Department of Physics, University of Pavia, Via Bassi 6, Pavia 27100, Italy; ∥ Department of Chemistry, Biology and Biotechnology, University of Perugia and INSTM, Perugia 06123, Italy; ⊥ SKKU Institute of Energy Science and Technology (SIEST) Sungkyunkwan University, Suwon 440-746, Korea

## Abstract

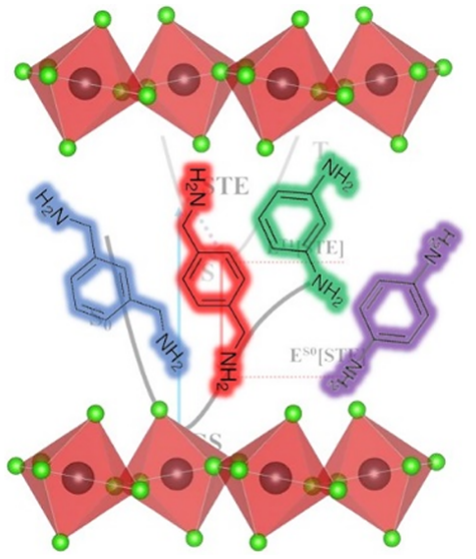

The development of broadband emitters based on metal halide perovskites (MHPs) requires the elucidation of structure–emission property correlations. Herein, we report a combined experimental and theoretical study on a series of novel low-dimensional lead chloride perovskites, including ditopic aromatic cations. Synthesized lead chloride perovskites and their bromide analogues show both narrow and broad photoluminescence emission properties as a function of their cation and halide nature. Structural analysis shows a correlation between the rigidity of the ditopic cations and the lead halide octahedral distortions. Density functional theory calculations reveal, in turn, the pivotal role of octahedral distortions in the formation of self-trapped excitons, which are responsible for the insurgence of broad emission and large Stokes shifts together with a contribution of halide vacancies. For the considered MHP series, the use of conventional octahedral distortion parameters allows us to nicely describe the trend of emission properties, thus providing a solid guide for further materials design.

Two-dimensional metal halide perovskites (2D MHPs) have recently attracted great attention for application as light-absorbing and/or light-emitting materials or as passivation layer on top of their 3D counterparts.^1−8^ The inherently layered nature of 2D MHPs with an A_2_BX_4_ chemical structure opens a tremendous construction kit made of A-site cations of various chemical building blocks, while the B-site and X-site typically stick with traditional metal (B = Pb, Sn) and halide ions (X = I, Br, Cl), respectively.^9−13^ While the optoelectronic properties are typically controlled by the inorganic scaffold, appropriate A-site cation engineering tailors the stability of MHPs and may modulate band alignment and their excitonic properties.^14−18^ A-site cations can be distinguished by their charge, with monoammonium and diammonium cations being of most relevance for the design of layered 2D MHPs.^11^ While a large variety of monoammonium cations has already been demonstrated, diammonium cations as organic spacers in 2D MHPs are systematically underexplored.^11,19−21^ Being ditopic ligands, such cations can directly interact with two separate inorganic layers. As for monoammonium cations, both linear and cyclic cations have been considered recently, suggesting a wide structural variability in diammonium-based 2D MHPs.^22−25^ However, ditopic organic cations do not always lead to the formation of a layered perovskite structure but instead may also give rise to other topologies.

Linear diammonium cations of general formula NH_3_(CH_2_)_*m*_NH_3_^2+^ with even carbon-chain lengths (*m* = 4, 8, 10, 12) form *n* = 1 2D lead-halide perovskites, while those with an odd carbon chain length (*m* = 7) form 1D perovskitoid structures.^26^ Moreover, diammonium cations with fused aromatic rings only give rise to 2D MHPs when being able to tilt in order to form hydrogen bonds to the halides.^27^ In general, the tilting of diammonium cations seems to be of great importance, since aromatic cations, where the ammonium groups may have no degree of freedom, tend to form 1D perovskitoid motifs. This is the case for (4,4′-MDA)PbI_4_ (MDA = methylenedianilinium) and (1,4-PDA)PbI_4_ (PDA = phenylene-*p*-diammonium), while a similar but asymmetric diammonium cation *N*,*N*-dimethylphenylene-*p*-diamine (DPDA) forms a layered structure with not only iodide and bromide but also 1D perovskitoids (DPDA)_2_PbI_5_·I under different stoichiometric conditions.^24,27,28^ Layered 2D MHPs were further reported for (1,3-PDA)PbBr_4_, (1,4-PDA)PbBr_4_, and (1,4-XDA)PbBr_4_ (XDA = xylylenediammonium), together with (1,3-PDA)PbI_4_^29^ and (AEA)PbBr_4_ (AEA = 3-(2-ammonioethyl)anilinium).^30,31^ Heterocyclic diammonium cations such as 2,2′-biimidazolium, benzodiimidazolium, and 1,4-dimethylpiperazinium have also been reported, with the latter cation showing the 2D structure with *n* = 1 for bromide compounds and 1D motifs with iodide.^32,33^ A common feature of the 2D MHPs with ditopic cations is the relatively small interlayer distance due to their compact nature.^11^

2D MHPs incorporating diammonium cations not only show this rich and fascinating structural chemistry but also offer appealing functional properties for optoelectronic applications. Diammonium ligands have been considered in the photovoltaics field with, for example, the incorporation of propylenediammonium and trimethylenediammonium in FASnI_3_ leading to improved film morphology and optoelectronic properties.^34^*ortho-*, *meta-* and *para-*isomers of (phenylene)di(ethylammonium) iodide have been used as passivating layers in perovskite solar cells, boosting the efficiency and long-term stability.^35^ Recent reports showed improved charge transport properties using diammonium cations in 2D Dion-Jacobson (DJ) MHPs, likely caused by a decrease in the gap between the 2D MHPs with charge transport layers.^36^

Even more interestingly, broadband white light emission has been observed in (*N*-MEDA)PbBr_4_ (*N*-MEDA = *N*^1^-methylethane-1,2-diammonium) and (EDBE)PbX_4_ (EDBE = 2,2′-(ethylenedioxy)bis(ethylammonium); X = Cl, Br), as well as for its tin-based counterpart (EDBE)SnI_4_.^37,38^ Recently, broadband emission was also observed in 2D lead bromide perovskites with ditopic aromatic cyclic cations, likely being correlated to the level of octahedral distortion.^30,39^

Still, the spectrum of available 2D MHPs with diammonium spacers remains limited with respect to their monoammonium counterparts. In addition, ditopic cations have been neglected so far in 2D lead chloride perovskites with respect to their iodide and bromide counterparts. This hinders the comprehension of the role of structural degrees of freedom within the diammonium cations in both the resulting structural motifs (2D or 1D) and the functional properties. Understanding the latter is a fundamental prerequisite to design novel and tailored diammonium-based 2D MHPs.

To provide a concise picture on the role of tuning the diammonium cation and the halide in 2D MHPs, we synthesize novel low-dimensional lead chloride perovskites with four different aromatic diammonium cations (1,3-phenylenediammonium (1,3-PDA), 1,3-xylylenediammonium (1,3-XDA), 1,4-phenylenediammonium (1,4-PDA), and 1,4-xylylenediammonium (1,4-XDA)) and characterize their structural and optoelectronic properties in comparison with our previously synthesized 2D lead bromide compounds.^30^ 1,3-PDA, 1,4-PDA, and 1,4-XDA result in 2D lead chloride perovskites, whereas 1,3-XDA forms a 0D perovskitoid structure. Interestingly, we observe significant differences in the light emission upon changing the diammonium cations and the halides, ranging from sharp emission close to the absorption edge to broad, red-shifted light emission. Density functional theory (DFT) calculations demonstrate the correlation between the broad emission with the self-trapping of excitons inside the inorganic scaffold for the majority of 2D perovskites, while emission within the 0D perovskitoids (1,3-XDA)_2_PbCl_6_ originates from the organic spacers themselves. Sharp band-to-band emission is only observable for the (1,4-XDA)PbBr_4_ showing the lowest octahedral distortions, in which self-trapping of excitons is absent in our theoretical calculations. Our results reveal a direct relation between the structural and the optoelectronic properties of diammonium-based low-dimensional perovskites, providing design rules for novel 2D MHPs with tailored optoelectronic properties.

Single crystals of the four lead-chloride compounds with 1,3-PDA, 1,4-PDA, 1,3-XDA, and 1,4-XDA have been grown (see Experimental Section in the Supporting Information, SI) and characterized by single-crystal X-ray diffraction (chemical structure reports in Figure S1 of the Supporting Information, SI). The resulting crystal structures and chemical formulas are listed in Table 1. Note that 1,3-PDA and 1,4-PDA have been previously used in the preparation of chloride perovskites reporting only the crystal structure without any further characterization of optical or electronic properties.^25^ According to Table 1, the prepared compounds show different structure types, as depicted in Figure 1.

**Table 1:** Crystal Structure Data for A_*n*_PbCl_*m*_ samples (A = 1,3-PDA, 1,3-XDA, 1,4-PDA, 1,4-PDA)

sample	chemical formula	space group and volume (Å^3^)	*a*, *b*, *c* (Å)	α, β, γ (°)
(1,3-PDA)PbCl_4_	(1,3-C_6_H_10_N_2_)PbCl_4_	P2/*c*	20.486(1)	90
		monoclinic	8.4386(5)	92.735(6)
		1223.1(1)	7.0834(5)	90
(1,3-XDA)_2_PbCl_6_	(1,3-C_8_H_14_N_2_)_2_PbCl_6_	*P*2_1_/*c*	10.5995(7)	90
		monoclinic	13.8588(7)	102.518(7)
		1192.1(1)	8.3131(5)	90
(1,4-PDA)Pb_2_Cl_6_	(1,4-C_6_H_10_N_2_)Pb_2_Cl_6_	*P*2_1_/*c*	13.7789(8)	90
		monoclinic	7.8328(4)	105.622(6)
		777.53(8)	7.4805(4)	90
(1,4-XDA)PbCl_4_	(1,4-C_8_H_14_N_2_)PbCl_4_	*Pnma*	7.7006(3)	90
		orthorhombic	24.3807(9)	90
		1467.3(1)	7.8154(3)	90

**Figure 1:**
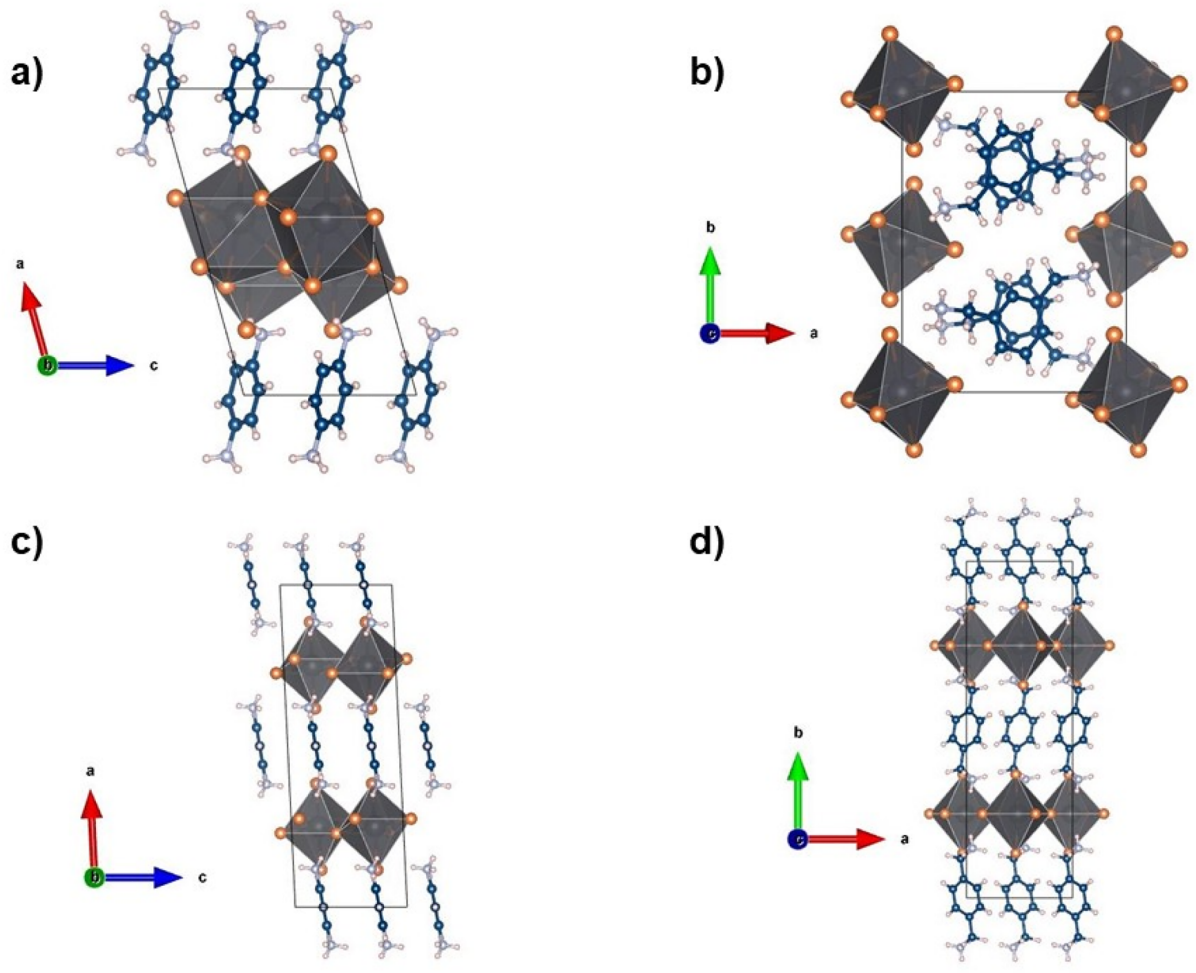
Crystal structures of (a) (1,3-PDA)PbCl_4_, (b) (1,3-XDA)_2_PbCl_6_, (c) (1,4-PDA)Pb_2_Cl_6_, and (d) (1,4-XDA)PbCl_4_.

(1,3-XDA)_2_PbCl_6_ does not have a layered structure but corresponds to a so-called 0D perovskitoid constituted by isolated octahedra. Notably, it is isostructural to (1,3-XDA)_2_PbBr_6_, suggesting that the 1,3-XDA cation may have a large tendency to form this topology.^30^ This structure type is probably not common with diammonium cations, even if other chloride-containing 0D systems have been reported in the past.^25^ (1,4-PDA)Pb_2_Cl_6_ shows a layered structure, with an inorganic layer made of face-sharing square antiprisms and a layer of organic cations located on the inversion center. The central Pb atom is eightfold coordinated with Pb–Cl distances ranging from 2.8029(18) to 3.3869(15) Å, creating a distorted square antiprism. The structure described in this work is in very good agreement with previous works, for which optical data were note reported.^25,40^ On the other hand, (1,3-PDA)PbCl_4_ and (1,4-XDA)PbCl_4_ crystallize as 2D Dion–Jacobson (DJ) phases with *n* = 1 comprising layers of PbCl_6_ octahedra separated by layers of organic cations. It is well-known that the interaction between the ammonium group and the octahedral framework deeply affects the structure and properties of layered perovskites.^9,10^ A useful parameter to explore this effect is the penetration depth, which is the distance between the N atom of the amino group and the plane of the terminal halides.^20^ The NH_3_^+^ penetration influences both the interoctahedral, in terms of the X–Pb–X angle, and intraoctahedral distortion parameters, in terms of the octahedral elongation length (⟨λ_oct_⟩) and bond angle variance (σ_oct_^2^).^30,41^ In particular, considering for comparison the bromide- and chloride-containing phenylamines reported so far, some general trends can be envisaged (Figure 2a). With the same organic cation, the penetration depth increases from the chloride to the bromide perovskites, probably as a consequence of the different strength of the hydrogen bond.^20,42,43^ The position and length of the substituents seem to play a role: 1,3-PDA cations have a shorter depth of penetration, most likely because of the steric hindrance of the relatively close substituents, while the 1,4-PDA and 1,4-XDA cations have larger penetration and more regular octahedral layers. Among them, 1,4-PDA gives rise to the largest tilting, i.e., the smallest X–Pb–X angle, since it is short and rigid and has less degrees of freedom, while the 1,4-XDA cation has longer substituents that can more easily interact with the halide.

**Figure 2:**
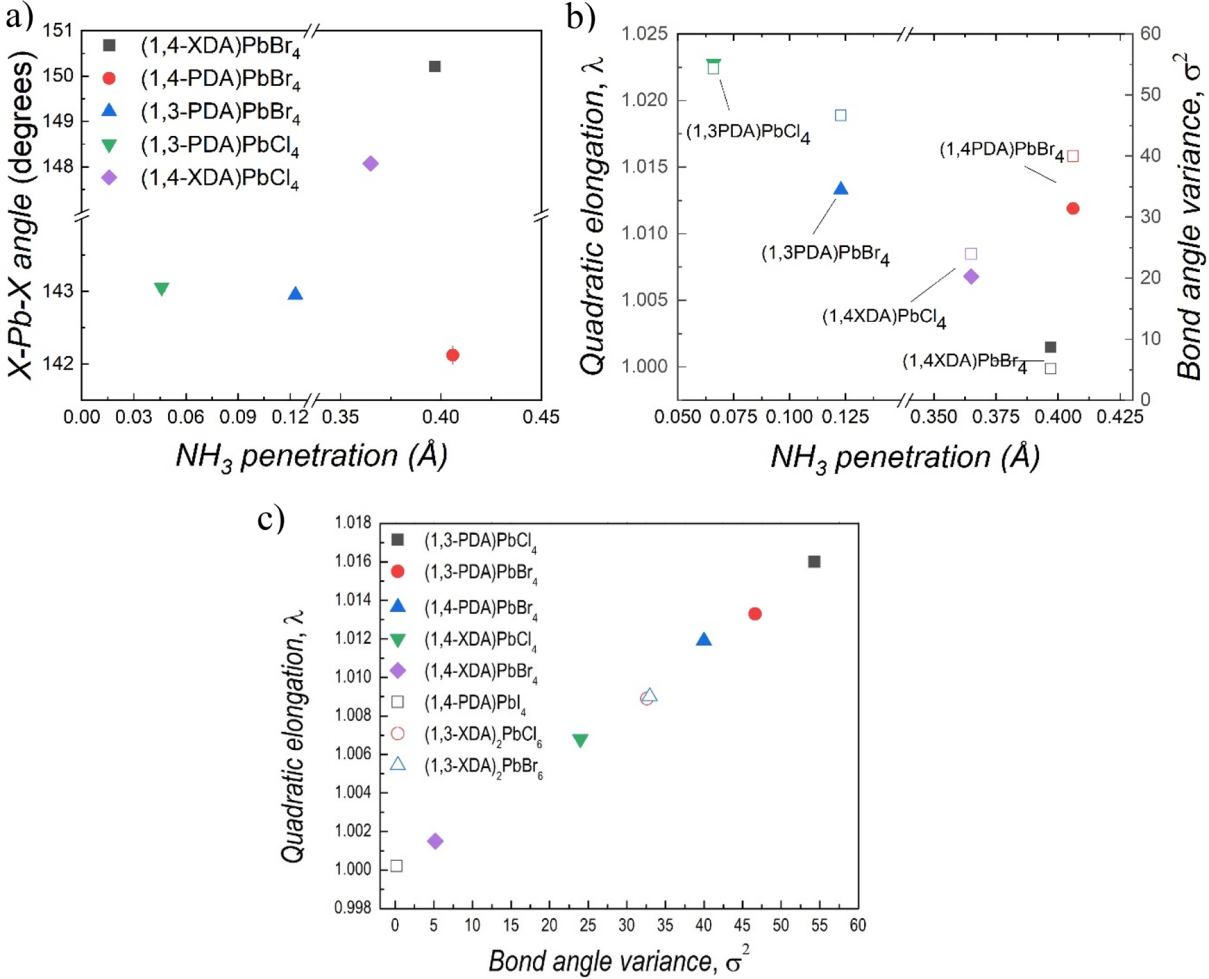
(a) X–Pb–X angle and (b) octahedral elongation and bond angle variance as a function of NH_3_ penetration for chloride and bromide compositions crystallizing in 2D perovskites. (c) Octahedral elongation as a function of bond angle variance. PDA = phenylenediammonium and XDA = xylylenediammonium.

Similar but opposite trends can also be seen in the intraoctahedral distortion parameters (Figure 2b). In fact, the 1,3-PDA cations have small NH_3_ penetration but give rise to large distortions within the octahedra. Among the 1,4-cations, the rigidity of 1,4-PDA again seems to affect the PbX_6_ octahedra, inducing a distortion close to that found for the 1,3-PDA cations. On the other hand, the more flexible 1,4-XDA cations allow for more regular octahedra. When the same organic cation is involved, the distortion within the octahedra increases with the hardness of the halide from Br to Cl, as predicted by first-principles calculations^44^ and shown by local structure studies.^45,46^ Finally, in order to gain further insight into the role of the flexibility of the organic cation, we extend the analysis of the intraoctahedral distortion to include all the compounds with available crystal structures, even those not showing a layered perovskite structure (Figure 2c). It is worth noting that the smallest distortion is shown by (1,4-PDA)PbI_4_, where symmetry constraints together with the edge sharing motif present in the structure give rise to regular lead iodide octahedra.^24^ Then, the XDA cations have an increasing distortion, with 1,3-XDA cations generating larger but very close distortions, supporting the idea that the 0D arrangement is mostly due to the type of organic cation. The more rigid PDA cations, and in particular the 1,3-cations, induce the largest distortions within the octahedra. Furthermore, as already pointed out, the distortion increases with the hardness of the halide passing from iodide to bromide and chloride. Based on these results, it is clear that among the low-dimensional perovskites including the four ditopic cations considered in this work, the chloride samples show the highest degree of distortion, which may have a relevant effect on the optical properties. The stability of the prepared samples has been assessed by collecting XRD patterns after 1 week of laboratory air exposure, confirming the very good stability when compared to the calculated patterns from the single crystal structures (Figure S2).

The optical properties of the four chloride-based samples are investigated by room temperature absorption and PL measurements, which are reported in Figure 3a. The absorbance (solid lines), calculated from the reflectivity of powdered single crystals, shows an absorption edge for the four samples in the range between 300 and 360 nm, an expected value for 2D lead chloride perovskites.^11,47−50^ From these measurements, the band gaps of the four samples have been determined: 3.53 eV for (1,3-PDA)PbCl_4_, 3.92 eV for (1,4-PDA)Pb_2_Cl_6_, 3.87 eV for (1,3-XDA)_2_PbCl_6_, and 3.51 eV for (1,4-XDA)PbCl_4_. A clear blue-shift in the band gap is observed for nonperovskite structures, which is due to confinement effects in the electronic structure of the inorganic scaffold, while for (1,3-PDA)PbCl_4_ and (1,4-XDA)PbCl_4_ the close values confirm that the main contribution of halide and lead in the band edges remain unchanged upon exchange of the cation, as confirmed by our DFT calculations (see Figures S3 and S4 in the Supporting Information).

**Figure 3:**
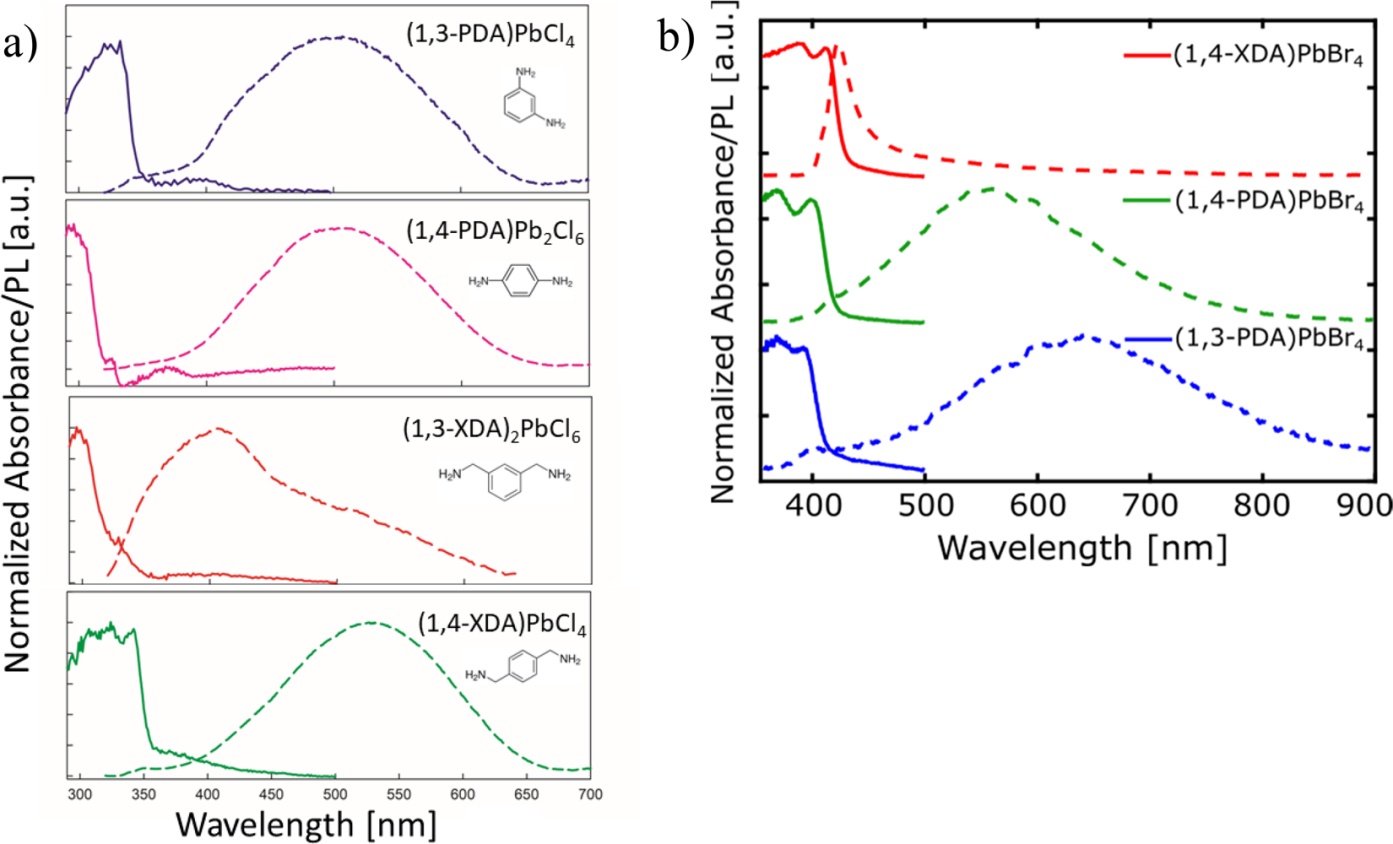
(a) Absorption (solid lines) and PL (dashed lines) spectra of (1,3-PDA)PbCl_4_ (blue line), (1.4-PDA)Pb_2_Cl_6_ (purple line), (1,3-XDA)_2_PbCl_6_ (red line), and (1,4-XDA)PbCl_4_ (green line) at RT. (b) Absorption (solid lines) and PL (dashed lines) spectra of (1,4-XDA)PbBr_4_ (red line), (1.4-PDA)PbBr_4_ (green line), and (1,3-PDA)PbBr_4_ (blue line) at RT.^30^ All spectra were acquired using an excitation wavelength, λ_ex_, of 300 nm.

The static PL (dashed lines) in Figure 3a, measured under an excitation at 300 nm, commonly shows a substantially red-shifted broad emission for (1,3-PDA)PbCl_4_, (1,4 -PDA)Pb_2_Cl_6_, and (1,4-XDA)PbCl_4_, extending in most of the visible spectrum, with peaks centered at 503, 506, and 525 nm, respectively, and fwhm’s around 200 nm. For (1,3-XDA)Pb_2_Cl_6_, on the other hand, an asymmetric PL with a peak centered around 410 nm, followed by a longer tail, is found. Moreover, for the previous three samples, a very large Stokes shift of more than 200 nm is observed, while for the latter, (1,3 XDA)Pb_2_Cl_6_, the Stokes shift is around 100 nm. Broadband emission has been observed in few chloride perovskites including monoammonium cations and in just one composition including a diammonium cations, namely 3-aminopyrrolidinium (displaying a (110)-oriented 2D perovskite structure).^31,48−51^ Finally, the role of the halide in the distortion and, in turn, in the emission behavior can be isolated by comparing the bromide and chloride analogues that form 2D DJ perovskites. The spectroscopic measurements carried out previously by our group on the bromide analogues, namely (1,3-PDA)PbBr_4_, (1,4-PDA)PbBr_4_, and (1,4-XDA)PbBr_4_, are reproduced in Figure 3b to allow a direct comparison.^30^ Both bromide and chloride samples including 1,3-PDA display a broadband emission, in agreement with the high distortion induced by this cation. On the other hand, a transition from narrow to broad emission is observed when moving from (1,4-XDA)PbBr_4_ to (1,4-XDA)PbCl_4_. Looking at the PL spectra, the majority of investigated perovskites shows a substantial Stokes shift. The only exception is (1,4-XDA)PbBr_4_, showing a sharp PL peak at 420 nm and a broad but less intense peak centered at around 600 nm.

In the literature, broad emission is often attributed to either self-trapped excitons or emission from defect states, typically halide vacancies, while a clear connection to their structural properties is still under debate.^51−54^ Our experimental PL data of a broad range of materials with similar structural moieties may allow for further insight into the nature of the broad emission features in 2D MHPs. DFT calculations are performed to rationalize the origin of the broad emission, distinguishing emission from self-trapped excitons (STEs) and halide vacancies, V_X_. The energy of STE emission is calculated by the difference between the energies of the triplet excited state and singlet ground states, *E*^T1^(STE) and *E*^S0^(STE), respectively, calculated at the equilibrium geometry of the STE; see the schematic representation in Figure 4.
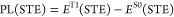


**Figure 4:**
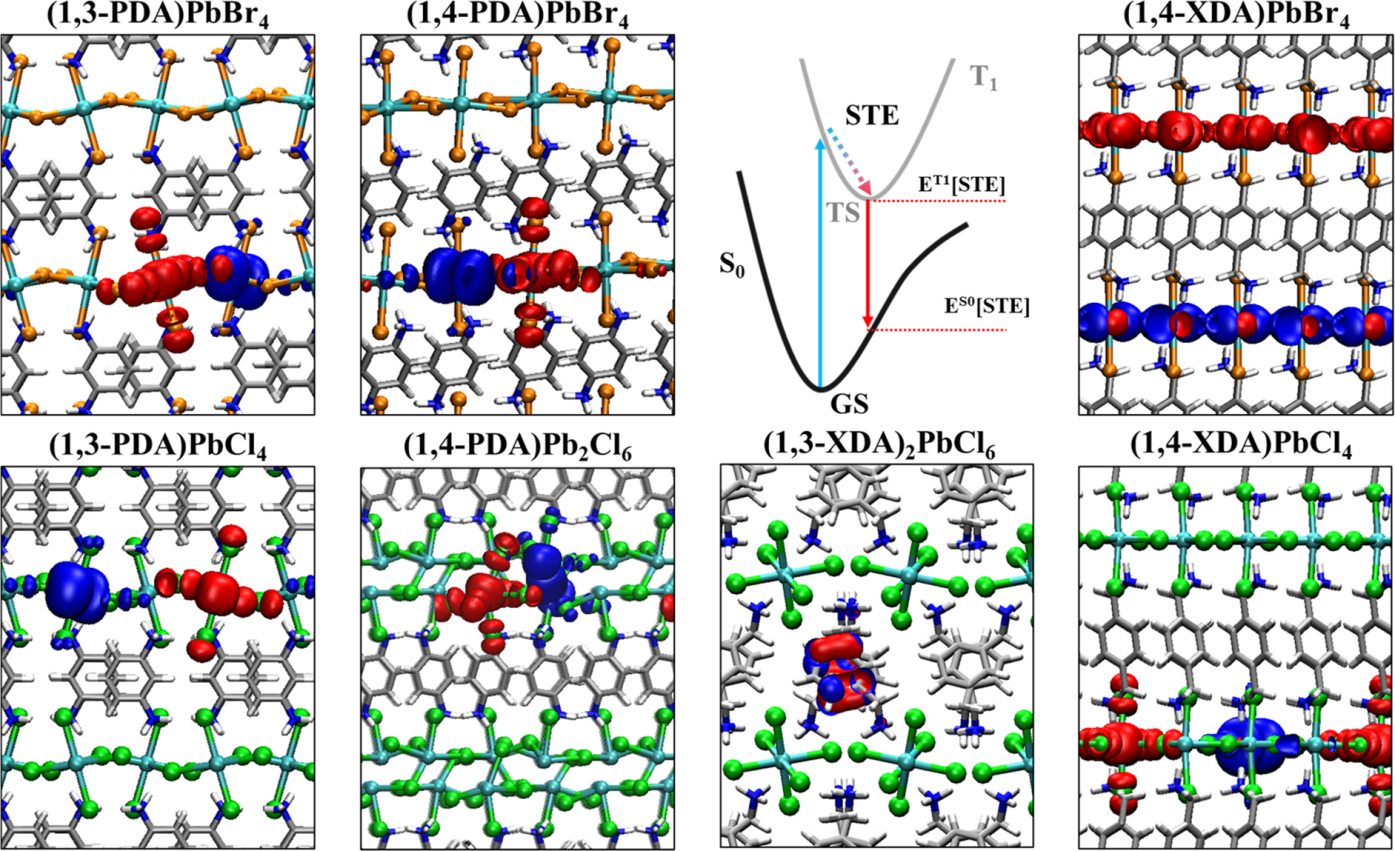
Visualization of electron (blue) and hole (red) Kohn–Sham (KS) orbitals for diammonium-based low dimensional MHPs after geometry relaxation in the excited triplet state. The Jablonski diagram in the upper row visualizes the formation mechanism of self-trapped excitons and the red-shifted PL emission from the STE to the potential energy surface of the ground state. (1,4-XDA)PbBr_4_ shows delocalized states along the inorganic scaffold. In (1,3-XDA)_2_PbCl_6_, the triplet state localizes within the 1,3-XDA cation. Self-trapping of excitons is observed in all remaining compounds.

We note that geometry optimization of the system using triplet spin multiplicity imposes an electron and a hole in the conduction band and in the valence band, respectively. This strategy was shown to result in a fair approximation to capture PL emission energies from photoexcited electron–hole pairs and further accounts for the higher stability of the triplet excited state after charge relaxation.^51,55,56^

Emission from halide vacancies is given by
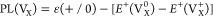
with thermodynamic ionization level ε(+/0), the energy of the positively charged supercell at V_X_^+^ and V_X_^0^ equilibrium geometry, in line with previous studies.^51,57,58^ Further computational details are given in the Supporting Information.

We obtain an excellent agreement between the theoretical values of PL emission energies from STE and the experimental ones for a large set of perovskites, in particular (1,3-PDA)PbBr_4_, (1,4-PDA)PbBr_4_, (1,3-PDA)PbCl_4_, (1,4-PDA)Pb_2_Cl_6_, (1,3-XDA)_2_PbCl_6_, and (1,4-XDA)PbCl_4_, see Table 2. Emission from halide vacancies shows comparable energy values (Table 2), likely contributing to the broad emission of all investigated perovskites. Note that for (1,3-XDA)_2_PbCl_6_ STE localizes within the organic cations, as the electronic states of the carbon moieties dominate the conduction band edge of the given 0D perovskitoid, see Figure S4c and Figure S5 in the SI). In the remaining perovskites, STEs form within the inorganic lead halide scaffold, see Figure 4. Despite the structural similarity, (1,4-XDA)PbBr_4_ does not show STE formation. Self-trapping of excitons requires a certain distortion of the inorganic scaffold, which appears less likely due to the reduction of octahedral tilting in (1,4-XDA)PbBr_4_, see Figure 2. Comparing all bromide-based 2D MHPs, no substantial differences in the electronic structure in the electronic ground state is observed, see Figure S2. We consequently assign the sharp PL peak at 420 nm to band-to-band emission of delocalized excitons. Furthermore, the presence (absence) of STE may be attributed to a large (low) octahedral tilting, providing an indicator for a rapid screening of potential material candidates for broad emitters. Our results further suggest that the strong red-shift is dominated by differences in the singlet energies between the ground state and the STE geometry, causing a substantial relaxation upon emission, see Table 2.

**Table 2:** Emission Energies for Diammonium-Based Perovskites with Bromide and Chloride As Halide Ions^a^

						theory	
perovskite	PL emission	T_1_(@GS)-GS	S_0_(@TS)-GS	T_1_(@GS)- TS	ΔSOC	STE	V_x_^b^
(1,3-PDA)PbBr_4_	1.9	3.73	1.13	0.34	0.37	1.89	2.21/2.50
(1,4-PDA)PbBr_4_	2.2	3.76	1.01	0.14	0.51	2.10	2.11/2.40
(1,4-XDA)PbBr_4_	3.0^c^	3.67	0.06	0.03			1.76/1.76
(1,3-PDA)PbCl_4_	2.5	4.44	1.50	0.51	0.25	2.18	2.34/2.29
(1,4-PDA)Pb_2_Cl_6_	2.4	4.78	1.61	0.58	0.24	2.35	2.94/2.67
(1,3-XDA)_2_PbCl_6_	3.0	4.03	1.23	0.22		2.96^d^	2.77/2.69
(1,4-XDA)PbCl_4_	2.4	4.24	1.22	0.22	0.36	2.44	2.24/2.19

a Experimental PL energies are extracted from UV–vis spectra, and theoretical values are obtained from DFT calculations distinguishing emission from self-trapped excitons (STEs) and halide vacancies V_X_ (X = Br or Cl) in equatorial (eq) and in apical (ap) position. Energy differences in the singlet (S_0_) and triplet states (T_1_) are reported, with TS and GS representing the relaxed geometries in the triplet and singlet states, respectively; see Figure 4. T_1_(GS) represents the single-point energy with triplet spin multiplicity at the singlet ground state geometry; S_0_(@TS) gives the single-point energy with singlet spin multiplicity at the relaxed triplet state geometry TS. T_1_(@GS)-GS is the vertical excitation energy, T_1_(@GS)-TS is the energy difference due to the relaxation of the system in the triplet state after photoexcitation, S_0_(@TS)-GS represents the energy difference due to relaxation of the singlet state geometry after emission, and Δ*S*OC represents the correction term to account for spin-orbit coupling effects due to the heavy Pb ions. Theoretical band gap energies are summarized in Table S2 and Supporting Information, for completeness.

b Refers to equatorial and apical vacancies, respectively

c Sharp emission peak due to band-to-band emission; no self-trapped excitons observed.

d Emission from 1,4-XDA cation.

Based on our results, we may hypothesize on the nature of broad emission when having different spectral features. The presence of a sharp emission peak is due to the band-to-band emission of delocalized excited states. Systems with sharp emission still may show broad emission features but with low intensity due to halide vacancies. Consequently, defect passivation may reduce the broad emission, in line with recent reports.^59^ Even if it is absent at room temperature, broad emission from defects may arise when decreasing the temperature due to imperfections in the lattice.^30,51^ In the case of STE formation, being favored by octahedral tilting, the sharp emission peak vanishes while a broad Stokes-shifted emission peak appears strongly. Emission from STE or from halide vacancies likely contributes to the broad emission features, while the formation of STEs is required to design broad emitters. Note that the presence of Stokes-shifted broad emission in the absence of a STE would require an enormous defect density. Considering the relatively sharp absorption edges, see Figure 3, halide vacancies alone may not cause, but certainly contribute to, the experimentally observed broad emission Stokes-shifted emission features. Note that we cannot fully distinguish the contributions from defects and STE emission. The latter aspect requires further investigations, likely by analyzing the dynamics of the PL features at different temperatures and defect concentrations by use of, for example, transient PL.^30^ Finally, we want to point out that the simple structural parameters σ^2^ and λ_oct_, reported in Figure 2 for the present samples, are excellent descriptors for an efficient screening of potential candidates for broad emission, in line with previous reports.^51,60,61^

Herein, we reported a series of novel low-dimensional lead chloride perovskites including four different monoammonium cations, namely, (1,3-PDA), (1,3-XDA), (1,4-PDA), and (1,4-XDA). Their crystal structures, elucidated by single-crystal X-ray diffraction, allowed the expansion of the actual comprehension of the role of the ditopic cation and halide nature on the distortion of the inorganic framework and their light emission properties, including in this correlation bromide analogues previously synthesized by our group.^30^ Based on this solid structure–property correlation, we provide tuning strategies to move from narrow emission, with a small Stokes shift, to broadband emitters with Stokes shifts of the order of 150–200 nm. The mechanism underpinning the different natures of the emission has been elucidated by DFT calculations. Narrow emission has been correlated to the band-to-band emission of delocalized excited states with low-intensity broad emission caused by halide vacancies. On the other hand, broadband emission has been unambiguously associated with the STE formation, in turn closely related to octahedral tilting, with a possible minor contribution to these spectral features of halide vacancies which, however, cannot account, alone, for the observed broad emission. Based on this extended combined experimental and computational modeling work, we conclude that, for bromide and chloride low-dimensional perovskites including a rigid ditopic cation, the description, and therefore modulation, of their emission properties can be realized by playing with the σ^2^ and λ_oct_ structural parameters. The present paper provides a solid base for the future design of broadband emitters, which are triggering huge interest due to their potential use as single emitters in optoelectronic devices.

## Supporting Information Available

Chemical structures of the diamines used to prepare the samples, projected density of states for all the compounds, LUMO molecular orbital representation for (1,3-XDA)_2_PbCl_6_, and materials and methods section including computational modeling details(PDF)
