# Supplementary material for: Origin of Broad Emission Induced by Rigid Aromatic Ditopic Cations in Low-Dimensional Metal Halide Perovskites
Source: J Phys Chem Lett. 2023 Aug 28;14(35):7860–8. doi: 10.1021/acs.jpclett.3c01872 (PMC10494231; doi:10.1021/acs.jpclett.3c01872)
Supplement: Supplementary file 1 — jz3c01872_si_001.pdf [file jz3c01872_si_001.pdf]

SUPPORTING INFORMATION

Origin of Broad Emission Induced by Rigid Aromatic Ditopic Cations in Low-Dimensional Metal Halide Perovskites

Marta Morana,[a] Waldemar Kaiser,[b,] Rossella Chiara,[a] Benedetta Albini,[c] Daniele Meggiolaro,[b] Edoardo Mosconi,[b] Pietro Galinetto,[c] Filippo De Angelis,[b,d,e] Lorenzo Malavasi[a,*]*

[a]Department of Chemistry and INSTM, University of Pavia, Via Taramelli 16, Pavia, 27100, Italy

[b]Computational Laboratory for Hybrid/Organic Photovoltaics (CLHYO), Istituto CNR di Scienze e Tecnologie Chimiche "Giulio Natta" (CNR-SCITEC), 06123 Perugia, Italy

[c]Department of Physics, University of Pavia, Via Bassi 6, Pavia, 27100, Italy

[d]Department of Chemistry, Biology and Biotechnology, University of Perugia and UdR INSTM, 06123 Perugia, Italy

[e]SKKU Institute of Energy Science and Technology (SIEST) Sungkyunkwan University, Suwon, Korea 440-746.

Corresponding Authors

Waldemar Kaiser, email: waldemar.kaiser@scitec.cnr.it;

Lorenzo Malavasi, email: lorenzo.malavasi@unipv.it; tel. +39 382 987921

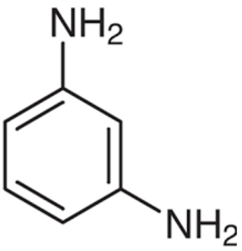

1,3-phenylenediammonium (1,3-PDA)

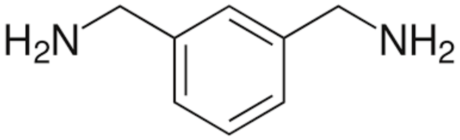

1,3-xylylenediamimonium (1,3-XDA)

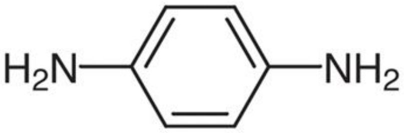

1,4-phenylenediammonium (1,4-PDA)

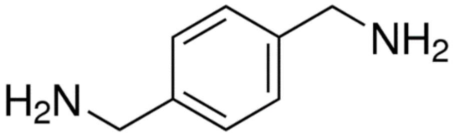

1,4-xylylenediamimonium (1,4-XDA)

Figure S1. Chemical structure of the diamines used in for the synthesis of Pb-Cl perovskites.

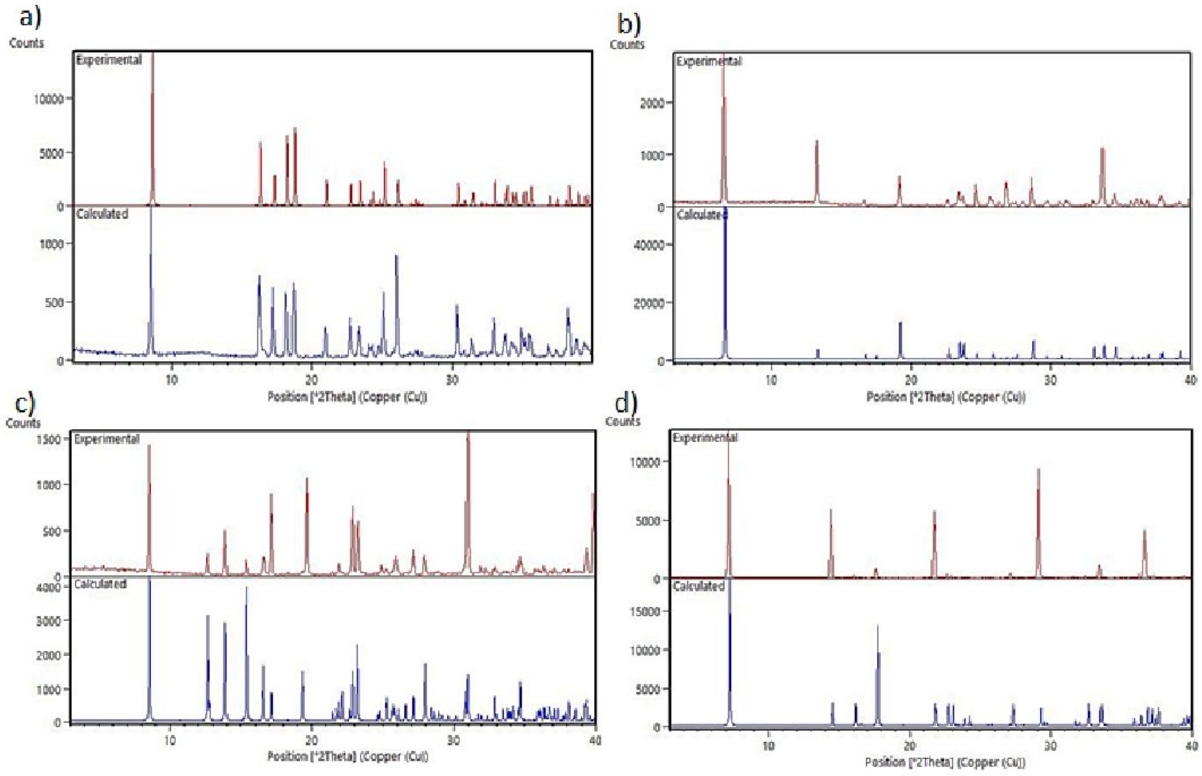

Figure S2. XRD powder patterns (red) of a) (1,3-PDA)$PbCl_4$, b) $(1,3\text{-}XDA)_2PbCl_6$, c) (1,4-PDA)Pb_2Cl_6, d) (1,4-XDA)$PbCl_4$ superimposed to the calculated patterns from SC-XRD (blue) after exposing the samples to laboratory air for one week.

Materials and methods

Synthesis

Single crystals of (1,3-PDA)$PbBr_4$, (1,4-PDA)$PbBr_4$, (1,3-XDA)$PbBr_4$ and (1,4-XDA)$PbBr_4$ were grown by dissolving a proper amount of lead(II) acetate powder in a large excess of 48% w/w aqueous HBr and 50% w/w aqueous H_3PO_2, heating the mixture to boiling point. After the solid dissolution, the stoichiometric amount of the solid diamine (liquid for the 1,3-XDA) was added. The crystals formation was obtained by a slow cooling down to room temperature at 2°C h^{-1}.

Single crystal and powder X-ray diffraction

Single crystal data collections (λ = 0.71073 Å) were performed using a Bruker D8 Venture with Cu and Mo microfocus X-ray sources and PHOTON II detector with Bruker APEX3 program. The Bruker SAINT software[1] was used for integration and data reduction, while absorption correction was performed using SADABS-2016/2[2]. Crystal structures (CCDC) were solved and refined using SHELXT 2014/5 and SHELXL 2018/3[3,4].

Photoluminescence and Absorption measurements

Photoluminescence: PL measurements were performed by means of a NANOLOG FL3-2iHR spectrofluorometer, Horiba Scientific, equipped with a 450W Xenon lamp as excitation source and an iHR320 triple-grating turret spectrometer as excitation monochromator. A single channel photomultiplier tube was used as detector.

Absorption: RS spectra were acquired in the wavelength range 300-800 nm directly on the powders by using a Jasco V-750 spectrophotometer, equipped with an integrating sphere (Jasco ISV-922).

Computational Details

Geometry optimization of all structures, both in the ground state and in triplet state, have been performed using the freely available CP2K software package, using the hybrid exchange-correlation functional PBE0 with α=0.25, including van der Waals interactions with the DFT-D3 scheme with Becke-Johnson damping.[5–8] Kohn-Sham orbitals are expanded in a double-zeta basis set (DZVP-MOLOPT) in combination with the norm-conserving Goedecker-Teter-Hutter (GTH) pseudopotentials, and a cutoff of 300 Ry for expansion of the electron density in plane waves.[9,10] The auxiliary density matrix method with the cFIT auxiliary basis set was applied to accelerate the optimization of ionic positions within hybrid functional calculations.[11]

Emission energies of the optimized geometries were refined using the PBE0 functional with $\alpha = 0.25$, including spin-orbit coupling (SOC), and DFT-D3 dispersion corrections within the Quantum Espresso package to correct for spin-orbit coupling effects due to the heavy Pb ions.[5,12] We used full relativistic norm-conserving pseudopotentials (Pb, 22 electrons, $5s^2$, $5p^6$, $5d^{10}$, $6s^2$, $6p^2$; Cl, 7 electrons, $3s^2$, $3p^5$; Br, 7 electrons, $4s^2$, $4p^5$; N, 5 electrons, $2s^2$, $2p^3$; C, 4 electrons, $2s^2$, $2p^2$; H, 1 electron, $1s^1$) with a cutoff on the wave functions of 40 Ry and 80 Ry on the Fock grid.

Defect formation energies (DFE) and thermodynamic ionization levels (TIL) were calculated as follows:

$$\mathrm{DFE}[\mathrm{X}^q] = \mathrm{E}[\mathrm{X}^q] - \mathrm{E}[\mathrm{prist}] - \sum_i \mathrm{n}_i\mu_i + \mathrm{q}(\mathrm{E}_{\mathrm{VBM}} + \mathrm{E}_\mathrm{F}) + \mathrm{E}^q_{\mathrm{corr}} \quad (1)$$

$$\mathrm{TIL}[q/q'] = \frac{\mathrm{DFE}[\mathrm{X}^q] - \mathrm{DFE}[\mathrm{X}^{q'}]}{q' - q} \quad (2)$$

where $E[X^q]$ is the energy of the defective supercell with defect X in charge state q, $E[prist]$ is the energy of the pristine supercell, n_i and μ_i are the number and chemical potential of the added and subtracted species, respectively, E_{VBM} and E_F are the valence band energy and the Fermi energy, respectively, and E^q_{corr} are electrostatic potential corrections due to the finite size of the supercell.[13,14] Electrostatic finite-size effects have been accounted for using the Freysoldt-Neugebauer-Van de Walle approach as implemented in the sxdefectalign code.[15]

Static dielectric constants were obtained using the density functional perturbation theory (DFPT) in the Vienna ab initio simulation package (VASP), with PBE exchange-correlation functional and the projector-augmented wave (PAW) method using a 300 eV plane-wave cutoff and converged k-space sampling for each compound based upon a tightly converged electronic wavefunction (within 10^{-8} eV).[16–19]

Table S1. Static dielectric constants for the bromide and chloride perovskite compounds from DFPT based on the PBE level of theory.

**Compound**	**Static Dielectric Constant**
(1,3-PDA)$PbBr_4$	14.0
(1,4-PDA)$PbBr_4$	10.9
(1,4-XDA)$PbBr_4$	8.1
(1,3-PDA)$PbCl_4$	12.7

(1,3-PDA)Pb_2Cl_6	12.6
(1,3-XDA)$_2$$PbCl_6$	6.2
(1,4-XDA)$PbCl_4$	6.0

Additional Plots

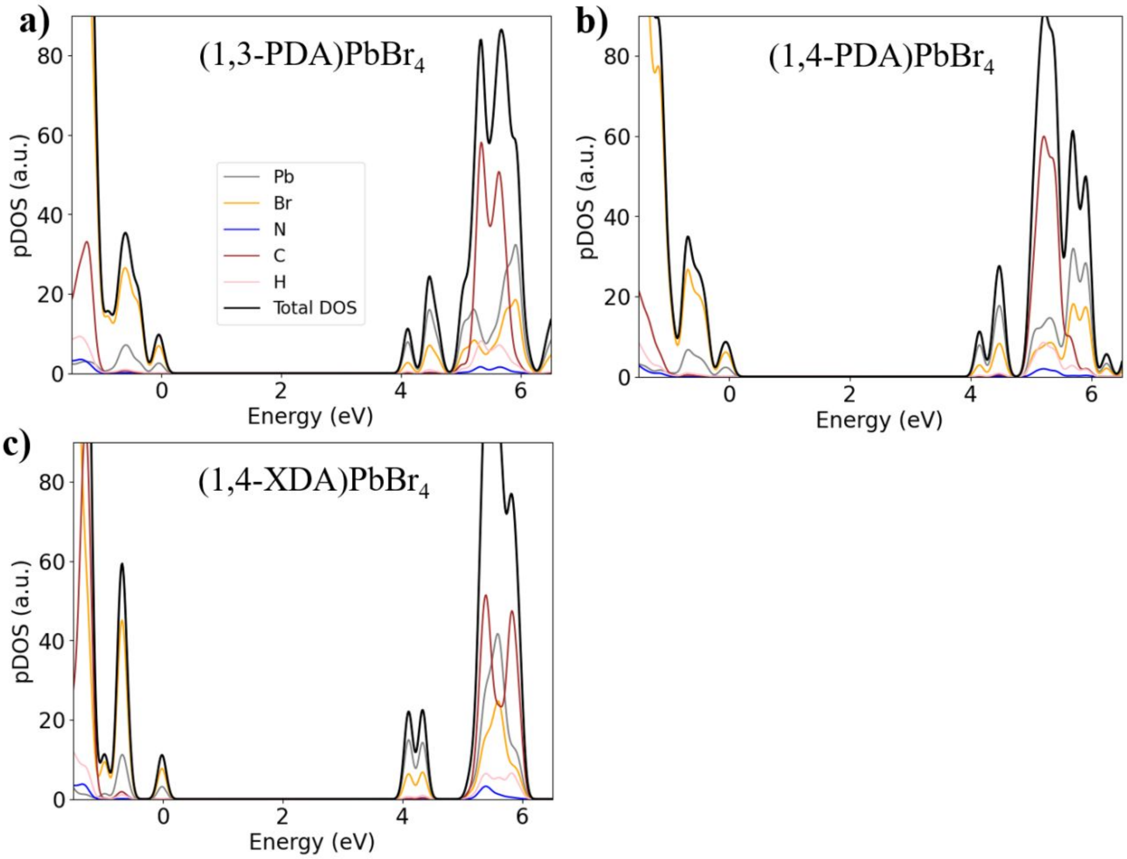

Figure S3. Projected density of states (pDOS) for investigate bromine-based 2D perovskites in the electronic ground state at PBE0 level of theory with α=0.25. The color code for all curves is given in the legend of panel a.

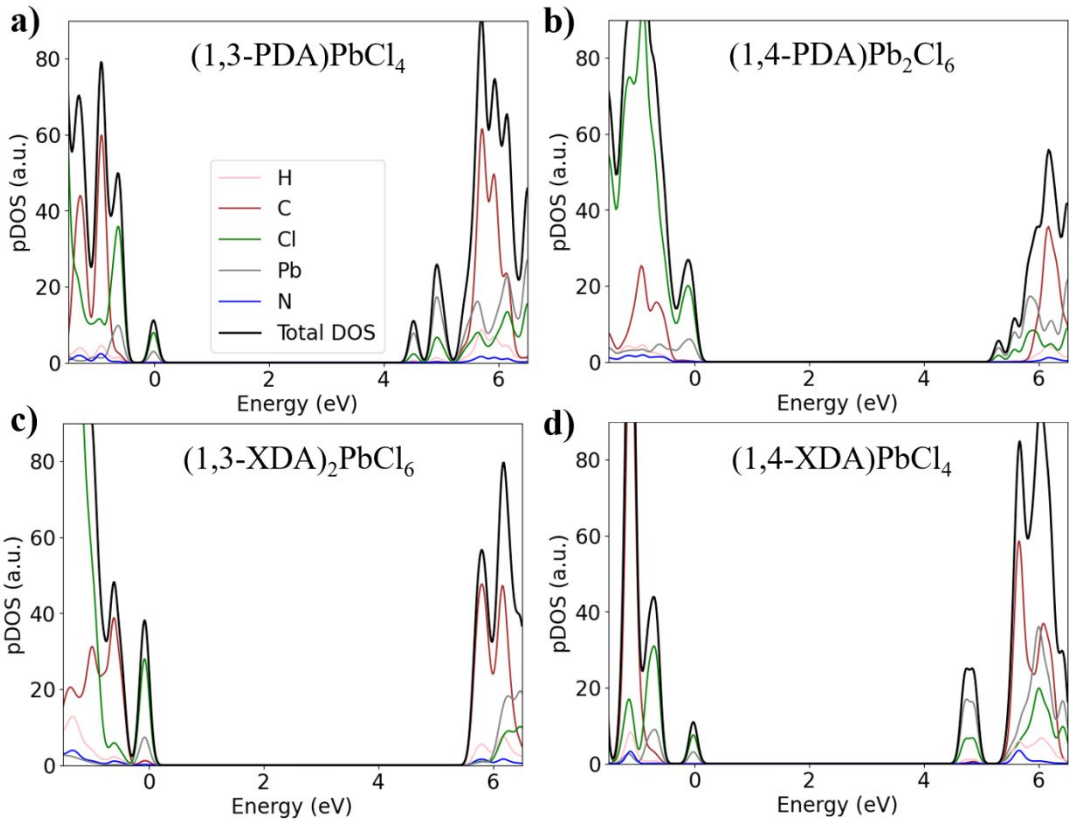

Figure S4. Projected density of states (pDOS) for investigate chlorine-based perovskites in the electronic ground state at PBE0 level of theory with α=0.25. The color code for all curves is given in the legend of panel a.

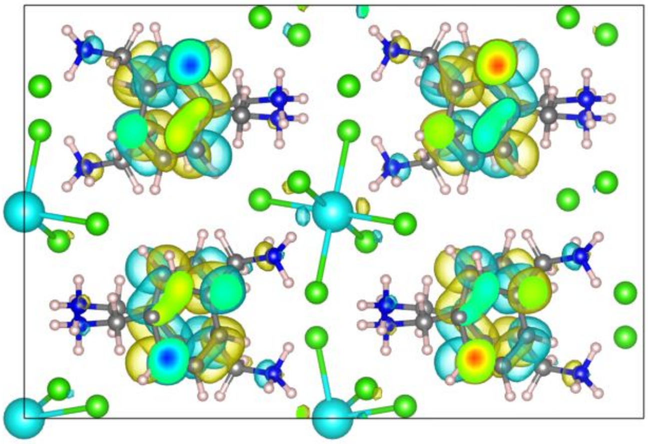

Figure S5. Visualization of lowest unoccupied molecular orbital (LUMO) for the 0D-perovskitoid (1,3-XDA)$_2$PbCl$_6$. The LUMO is delocalized along the 1,3-XDA cations, as expected from the pDOS, Figure S1c.

Table S2. Experimental band gap and theoretical band gap energies calculated on the PBE0 level of theory in units of eV.

**Perovskite**	**Exp. gap**	**Theor. gap**
(1,3-PDA)$PbBr_4$	~3	4.11
(1,4-PDA)$PbBr_4$	~3	4.14
(1,4-XDA)$PbBr_4$	~3	4.09
(1,3-PDA)$PbCl_4$	3.53	4.53
(1,4-PDA)Pb_2Cl_6	3.92	5.30
(1,3-XDA)$_2PbCl_6$	3.87	5.66
(1,4-XDA)$PbCl_4$	3.51	4.69

References

(1) *Bruker. SAINT*; Bruker AXS Inc., 2015.
(2) *Bruker. SADABS*; Bruker AXS Inc.: Madison, WI, USA, 2016.
(3) Sheldrick, G. M. SHELXT – Integrated Space-Group and Crystal-Structure Determination. *Acta Cryst A* **2015**, *71* (1), 3–8. https://doi.org/10.1107/S2053273314026370.
(4) Sheldrick, G. M. Crystal Structure Refinement with SHELXL. *Acta Cryst C* **2015**, *71* (1), 3–8. https://doi.org/10.1107/S2053229614024218.
(5) Grimme, S.; Antony, J.; Ehrlich, S.; Krieg, H. A Consistent and Accurate *Ab Initio* Parametrization of Density Functional Dispersion Correction (DFT-D) for the 94 Elements H-Pu. *The Journal of Chemical Physics* **2010**, *132* (15), 154104. https://doi.org/10.1063/1.3382344.
(6) Kühne, T. D.; Iannuzzi, M.; Del Ben, M.; Rybkin, V. V.; Seewald, P.; Stein, F.; Laino, T.; Khaliullin, R. Z.; Schütt, O.; Schiffmann, F.; Golze, D.; Wilhelm, J.; Chulkov, S.; Bani-Hashemian, M. H.; Weber, V.; Borštnik, U.; Taillefumier, M.; Jakobovits, A. S.; Lazzaro, A.; Pabst, H.; Müller, T.; Schade, R.; Guidon, M.; Andermatt, S.; Holmberg, N.; Schenter, G. K.; Hehn, A.; Bussy, A.; Belleflamme, F.; Tabacchi, G.; Glöß, A.; Lass, M.; Bethune, I.; Mundy, C. J.; Plessl, C.; Watkins, M.; VandeVondele, J.; Krack, M.; Hutter, J. CP2K: An Electronic Structure and Molecular Dynamics Software Package - Quickstep: Efficient and Accurate Electronic Structure Calculations. *J. Chem. Phys.* **2020**, *152* (19), 194103. https://doi.org/10.1063/5.0007045.
(7) Adamo, C.; Barone, V. Toward Reliable Density Functional Methods without Adjustable Parameters: The PBE0 Model. *The Journal of Chemical Physics* **1999**, *110* (13), 6158–6170. https://doi.org/10.1063/1.478522.
(8) Grimme, S.; Ehrlich, S.; Goerigk, L. Effect of the Damping Function in Dispersion Corrected Density Functional Theory. *J. Comput. Chem.* **2011**, *32* (7), 1456–1465. https://doi.org/10.1002/jcc.21759.
(9) VandeVondele, J.; Hutter, J. Gaussian Basis Sets for Accurate Calculations on Molecular Systems in Gas and Condensed Phases. *The Journal of Chemical Physics* **2007**, *127* (11), 114105. https://doi.org/10.1063/1.2770708.
(10) Goedecker, S.; Teter, M.; Hutter, J. Separable Dual-Space Gaussian Pseudopotentials. *Phys. Rev. B* **1996**, *54* (3), 1703–1710. https://doi.org/10.1103/PhysRevB.54.1703.
(11) Guidon, M.; Hutter, J.; VandeVondele, J. Auxiliary Density Matrix Methods for Hartree−Fock Exchange Calculations. *J. Chem. Theory Comput.* **2010**, *6* (8), 2348–2364. https://doi.org/10.1021/ct1002225.
(12) Giannozzi, P.; Baroni, S.; Bonini, N.; Calandra, M.; Car, R.; Cavazzoni, C.; Ceresoli, D.; Chiarotti, G. L.; Cococcioni, M.; Dabo, I.; Corso, A. D.; Gironcoli, S. de; Fabris, S.; Fratesi, G.; Gebauer, R.; Gerstmann, U.; Gougoussis, C.; Kokalj, A.; Lazzeri, M.; Martin-Samos, L.; Marzari, N.; Mauri, F.; Mazzarello, R.; Paolini, S.; Pasquarello, A.; Paulatto, L.; Sbraccia, C.; Scandolo, S.; Sclauzero, G.; Seitsonen, A. P.; Smogunov, A.; Umari, P.; Wentzcovitch, R. M. QUANTUM ESPRESSO: A Modular and Open-Source Software Project for Quantum Simulations of Materials. *Journal of Physics: Condensed Matter* **2009**, *21* (39), 395502. https://doi.org/10.1088/0953-8984/21/39/395502.
(13) Freysoldt, C.; Grabowski, B.; Hickel, T.; Neugebauer, J.; Kresse, G.; Janotti, A.; Van De Walle, C. G. First-Principles Calculations for Point Defects in Solids. *Rev. Mod. Phys.* **2014**, *86* (1), 253–305. https://doi.org/10.1103/RevModPhys.86.253.
(14) Meggiolaro, D.; De Angelis, F. First-Principles Modeling of Defects in Lead Halide Perovskites: Best Practices and Open Issues. *ACS Energy Lett.* **2018**, *3* (9), 2206–2222. https://doi.org/10.1021/acsenergylett.8b01212.

(15) Freysoldt, C.; Neugebauer, J.; Van De Walle, C. G. Fully *Ab Initio* Finite-Size Corrections for Charged-Defect Supercell Calculations. *Phys. Rev. Lett.* **2009**, *102* (1), 016402. https://doi.org/10.1103/PhysRevLett.102.016402.

(16) Baroni, S.; De Gironcoli, S.; Dal Corso, A.; Giannozzi, P. Phonons and Related Crystal Properties from Density-Functional Perturbation Theory. *Rev. Mod. Phys.* **2001**, *73* (2), 515–562. https://doi.org/10.1103/RevModPhys.73.515.

(17) Kresse, G.; Furthmüller, J. Efficient Iterative Schemes for *Ab Initio* Total-Energy Calculations Using a Plane-Wave Basis Set. *Phys. Rev. B* **1996**, *54* (16), 11169–11186. https://doi.org/10.1103/PhysRevB.54.11169.

(18) Perdew, J. P.; Burke, K.; Ernzerhof, M. Generalized Gradient Approximation Made Simple. *Phys. Rev. Lett.* **1996**, *77* (18), 3865–3868. https://doi.org/10.1103/PhysRevLett.77.3865.

(19) Blöchl, P. E. Projector Augmented-Wave Method. *Phys. Rev. B* **1994**, *50* (24), 17953–17979. https://doi.org/10.1103/PhysRevB.50.17953.
